# Associations between carabid beetles and fungi in the light of 200 years of published literature

**DOI:** 10.1038/s41597-021-01072-w

**Published:** 2021-11-04

**Authors:** Gábor Pozsgai, Ibtissem Ben Fekih, Markus V. Kohnen, Said Amrani, Sándor Bérces, Dávid Fülöp, Mohammed Y. M. Jaber, Nicolai Vitt Meyling, Malgorzata Ruszkiewicz-Michalska, Walter P. Pfliegler, Francisco Javier Sánchez-García, Jie Zhang, Christopher Rensing, Gábor L. Lövei, Minsheng You

**Affiliations:** 1 State Key Laboratory of Ecological Pest Control for Fujian and Taiwan Crops, Institute of Applied Ecology, Fujian Agriculture and Forestry University, Fuzhou, 350002 China; 2 Joint International Research Laboratory of Ecological Pest Control, Ministry of Education, Fuzhou, 350002 China; 3 CE3C – Centre for Ecology, Evolution and Environmental Changes, Azorean Biodiversity Group and Universidade dos Açores, Angra do Heroísmo, 9700-042 Azores, Portugal; 4 Institute of Environmental Microbiology, College of Resources and Environment, Fujian Agriculture and Forestry University, Fuzhou, 350002 China; 5 Basic Forestry and Proteomics Research Center, College of Life Science, Fujian Provincial Key Laboratory of Haixia Applied Plant Systems Biology, Fujian Agriculture and Forestry University, Fuzhou, 350002 China; 6 Laboratoire de Biologie et de Physiologie des Organismes, Faculté des Sciences Biologiques, Université des Sciences et de la Technologie Houari Boumediène, BP 32 El Alia, Alger, 16111 Algeria; 7 Duna-Ipoly National Park Directorate, Költő u. 21, H-1121 Budapest, Hungary; 8 Juhász-Nagy Pál Doctoral School, University of Debrecen, Egyetem tér 1, H-4032 Debrecen, Hungary; 9 Department of Zoology, Plant Protection Institute, Centre for Agricultural Research, Nagykovácsi út 26-30, H-1029 Budapest, Hungary; 10 Fujian University Key Laboratory for Plant-Microbe Interaction, College of Plant Protection, Fujian Agriculture and Forestry University, Fuzhou, 350002 China; 11 Department of Plant and Environmental Sciences, University of Copenhagen, Thorvaldsensvej 40, 1871 Frederiksberg C, Denmark; 12 Department of Algology and Mycology Faculty of Biology and Environmental Protection, University of Łódź, Banacha 12/16, PL-90-237 Łódź, Poland; 13 Department of Molecular Biotechnology and Microbiology, University of Debrecen, Egyetem tér 1, Debrecen, H-4032 Hungary; 14 Fujian Provincial Key Laboratory of Insect Ecology, College of Plant Protection, Fujian Agriculture and Forestry University, Fuzhou, 350002 China; 15 Área de Biología Animal, Departamento de Zoología y Antropología Física, Facultad de Veterinaria, Universidad de Murcia, Murcia, 30100 Spain; 16 Department of Agroecology, Aarhus University, Flakkebjerg Research Centre, Forsøgsvej 1, DK-4200 Slagelse, Denmark

**Keywords:** Agroecology, Macroecology, Ecological networks

## Abstract

Describing and conserving ecological interactions woven into ecosystems is one of the great challenges of the 21^st^ century. Here, we present a unique dataset compiling the biotic interactions between two ecologically and economically important taxa: ground beetles (Coleoptera: Carabidae) and fungi. The resulting dataset contains the carabid-fungus associations collected from 392 scientific publications, 129 countries, mostly from the Palearctic region, published over a period of 200 years. With an updated taxonomy to match the currently accepted nomenclature, 3,378 unique associations among 5,564 records were identified between 1,776 carabid and 676 fungal taxa. Ectoparasitic Laboulbeniales were the most frequent fungal group associated with carabids, especially with Trechinae. The proportion of entomopathogens was low. Three different formats of the data have been provided along with an interactive data digest platform for analytical purposes. Our database summarizes the current knowledge on biotic interactions between insects and fungi, while offering a valuable resource to test large-scale hypotheses on those interactions.

## Abstract (summary)

**Table:** 

Measurement(s)	species associations
Technology Type(s)	digital curation
Factor Type(s)	associations between Carabidae and Fungi
Sample Characteristic - Organism	Carabidae • Fungi • Laboulbeniales
Sample Characteristic - Location	global

Machine-accessible metadata file describing the reported data: 10.6084/m9.figshare.14602770

## Background & Summary

One of the striking features of the Anthropocene is a rapid degradation of natural ecosystems^1,2^, and an alarming decline of many species, which ultimately may lead to extinctions^3–5^. Whereas conserving ecosystem functions is increasingly recognised as a vital need for humans^6–8^, the interspecific interactions underpinning these functions are poorly understood^9,10^. However, conserving such interactions can be particularly important when taxa providing high-value ecosystem services are involved^10,11^.

Ground beetles (Coleoptera: Carabidae) have been long known for their benefits in agroecosystems^12,13^. They play an important role in suppressing pests^14^, but several carabid species also consume seeds of herbaceous plants, making them a valuable asset for weed control as well^15^.

Fungi are also of vital significance in most of the world’s terrestrial ecosystems^16^. Mycorrhizal fungi improve nutrient uptake by a large range of plant species through intimate and specialised associations^17^, other fungi play a crucial role in decomposition^18^, and yet others are pathogens of both crops and pests in agroecosystems^19^. Fungal parasitism is one of the crucial agents of evolution^20^.

Fungi and carabids often co-occur, and they can potentially interact in many ways. The soil environment carabids often inhabit is a reservoir of fungal propagules where the beetles can feed on spores, hyphae or fruiting bodies^21^. They may also be responsible for dispersal of spores of certain fungi^22^. Several parasitic or entomopathogenic fungi are in an obligatory relationship with their beetle hosts^23^, therefore, the population decline of a ground beetle species could potentially lead to overlooked extinction cascades^24^. However, our knowledge of the fungal-carabid interactions is still limited concerning the frequency of these interactions and on how their exact nature affect the parties involved. Indeed, we do not even have a catalogue of the carabid-fungi interactions, and they have not yet been organized into a comprehensive database. Such a database would be of particular importance from an integrated pest management point of view because both fungi and carabids can deliver ecosystem services, but how their interactions, and potential synergies or antagonisms, influence the delivery of these services is poorly understood.

In order to have a detailed overview of the interactions between Carabidae and the fungal kingdom, we collated a database containing previously reported associations between these taxa. Carabid and fungal species involved in the interaction, the type of the interaction (e. g. parasitic, pathogenic, mutualistic, or trophic interactions), the location (country) the interaction was reported from, and the publication source combined with detailed notes to each questionable entry comprised one record. Publications available in printed formats only were either digitized and data were extracted using semi-automatic text-mining processes, or they were manually screened. We aimed at possible completeness, using a wide range of databases and search engines and several languages to cover most of the published literature.

Both ground beetle and fungal names were validated and their higher taxonomical classifications were also extracted. When it was possible, historical localities were converted to their current country names. The full bibliographical details were also stored in the database.

The database covers a time-period from 1793 to 2020, spans over all geographic sub-regions defined by the United Nations (*“UNSD — Methodology”*, unstats.un.org. Retrieved 2020–10–11) with recorded associations from 129 countries. Our effort yielded 3,378 unique associations in 5,564 records between 1,776 carabid and 676 fungal species. Although rapidly developing molecular methods have largely facilitated the mapping of complex interaction networks in ecological studies^25–27^, due to the historic nature of our dataset, most of the records rely on traditional taxonomical identification. Yet, 16 records were based purely on metabarcoding studies; comments linked to these associations clearly identify them.

**Fig. 1:**
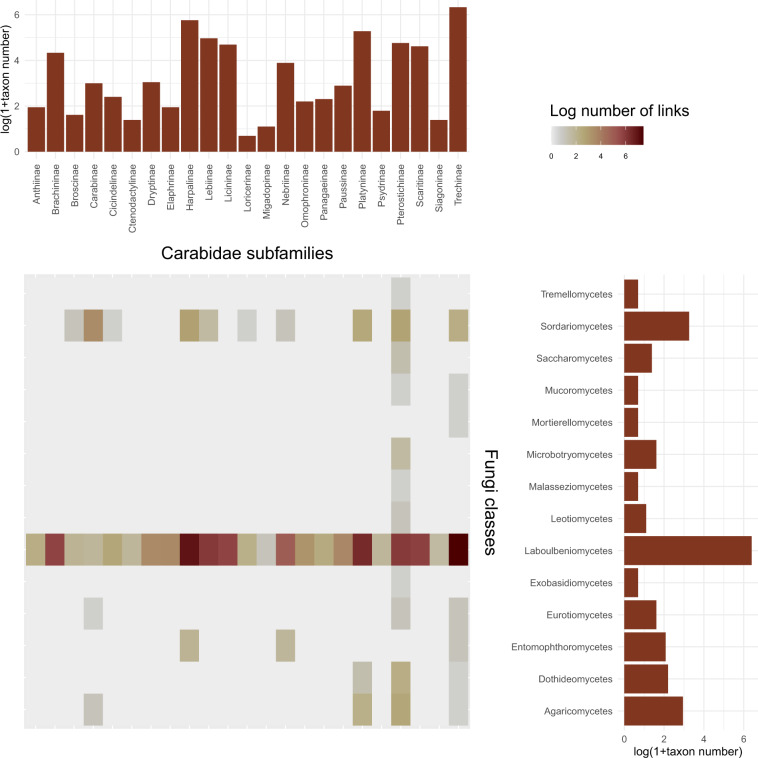
The number of unique associations between Carabidae subfamilies and fungal classes. Side bar plots show the number of species in each subfamily/class recorded in our dataset.

**Fig. 2:**
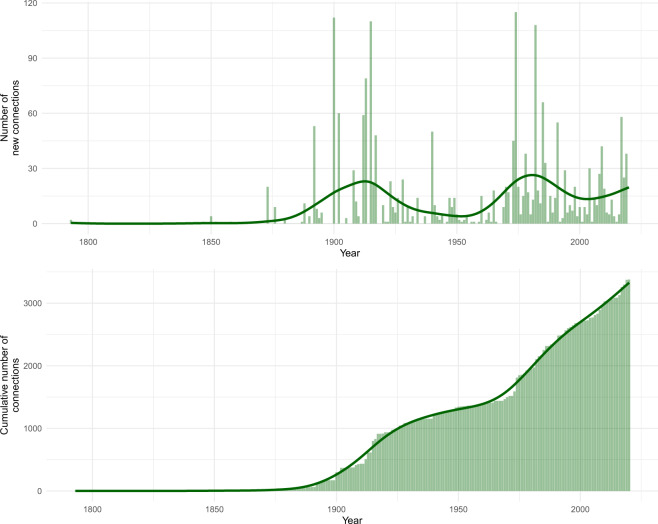
The number of recorded unique associations over time. Changes in the number of new records (**a**) and in the cumulative number (**b**) per year. Dark green lines indicate smoothed trends.

Whilst we found relatively few pathogenic interactions, a great diversity between ectoparasitic Laboulbeniales fungi and carabids was revealed (Fig. 1). Soft bodied, cave-dwelling members of the Trechinae subfamily were particularly prone to these parasitic infections. Little information was available on mutualistic relationships but the presence of *Yarrowia* yeast reported from the gut of several carabid species^28^ is probably beneficial for both parties. The data show two distinct peaks in publications registering new associations, in the early 19^th^ century and in the late 20^th^ century (Fig. 2a) but the steady increase in the cumulative number of associations (Fig. 2b) suggests that further research is required to fully resolve this association network. Although we believe that most of the data published so far were collected, data submission will remain open to researchers wishing to contribute.

## Methods

### Data compilation

#### Database scraping and literature search

**Fig. 3:**
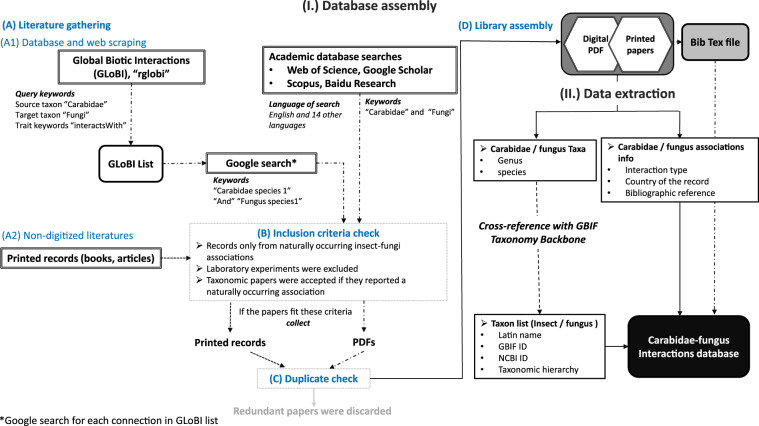
Flow chart of data collection of carabid-fungus associations from the literature.

Global Biotic Interactions is an open access database for finding species interactions^29^. As a starting point, we queried GloBI’s application programming interface (API) using the rglobi R package, with the target taxa set to “Carabidae” and “Fungi”. Since tiger beetles (Carabidae: Cicindelinae) are sometimes considered as a separate family, to avoid taxonomic discrepancies, we also searched “Cicindelidae” and “Fungi”. For this query, we used the application programming interface (API) provided by GloBI. We ran a series of further web searches targeting each registered interaction to find the first published sources and collect additional location data. We also mined academic databases and search platforms, including the Web of Science, Scopus, Google Scholar, and Baidu Research with the keyword sets of “Carabidae AND Fungi” or “Carabid* AND Fung*”. Additional searches were conducted using the words for ground beetles and fungi in Arabic, Chinese, Czech, Danish, Dutch, English, French, German, Hungarian, Japanese, Norwegian, Polish, Russian, Spanish, and Swedish languages (Fig. 3).

#### Data extraction

Each article in our literature database was individually scanned for associations and interactions and the information was mostly manually extracted, in most cases by collaborators who were native speakers of the language in which the publication was written. Articles reporting experiments of beetles being infected with fungi *in vitro* were excluded. However, reports of naturally occurring carabid-fungus associations, even if they were laboratory experiments, were included as well as taxonomic publications matching this criterion. Unique associations between carabid and fungal species were individually recorded from each country. Incomplete data with missing species names or country were also extracted and included in the primary dataset. These, however, were filtered out if more precise data were found from the same location or if an earlier, unreported location of a carabid-fungus associations was identified. Alongside each record, the literature source and the contributor who extracted the data were also recorded. This latter served quality control purposes only, and was not included in the final dataset.

### Data processing

#### Data checking, cleaning, and formatting

Data were first compiled into one large dataset then organized into separate tables (Online-only Table 1), which were then linked through unique identifiers in the MySQL interactions table. The integrity and uniqueness of each table were checked both programmatically and manually. Taxonomic names were cross-checked with the GBIF Taxonomy Backbone where old names were actualized, and the synonyms changed to currently valid names. When species names published in original articles could not be resolved to any valid taxon, only genera were recorded; if neither genus nor species could be validated, the record was omitted. For instance, *Brachinus “laevonaci”* was only recorded as genus *Brachinus*, while the record containing the “*Pardomis*” genus name was excluded. These records, despite not being published in the primary dataset, were kept for further investigations and will be added when new information regarding the taxonomic identity is identified. When species were split into sibling species, it was not possible to back-determine the valid taxon. This was particularly problematic in some recently revised fungal taxa, where the use of novel molecular methods substantially changed the taxonomy. These taxa were recorded as “sensu lato”.

Country names, and their geographic categorization were extracted from an altered version of the United Nations Geoscheme (*“UNSD — Methodology”*, unstats.un.org. Accessed 2020–10–11). We separated some politically joint but geographically distant regions, mostly islands, to better suit our biodiversity mapping purposes. For instance, Hawaii was handled separately from the U.S. and the Canary Islands were from Spain. A unique combination of one valid carabid, one valid fungal species, and the country where this association was recorded from was used as one data unit. A reference for this unique connection was joined with the data line. When different researchers reported the same association from the same country, the earliest published report was consistently used as a reference, while the later reports were omitted.

## Data Records

### Available formats and database structure

For our database to be easily accessible to those who are not familiar with MySQL databases, but at the same time keeping the dataset to be expandable, convertible, and queryable, we have provided the data in three different formats: a multi-table relational database in tables of comma separated values, one single MySQL file for the collection of those tables, and a single-file merged data table. All data files are available on *figshare*^30^: 10.6084/m9.figshare.14602479.v5.

#### Collection of data tables in comma separated values

Both data table collection and the importable SQL database consist of five tables: 1) collecting the unique associations/interactions between Carabidae and Fungi from a particular country, 2) listing carabid and fungal taxonomy, 3) listing the references used in the data collection, 4) geographic data for the countries from where the data were recorded, and 5) a metadata table for annotation (Online-only Table 1). The metadata table contains information on edits and uncertainties within the database. Unlike in the MySQL database, polygonal geometries for countries’ geographic information was not provided in these data tables.

#### Single data table

Data were also merged into a single.csv file containing 5,564 rows and 42 columns. In this table, column names are the same as in the other data storing methods, but columns were ordered according to their likely importance for the user. Thus, species names and GBIF IDs are presented first, followed by the association type, country, and the country code. Next, the unique identifier of the reference source, and the human-readable references follow (Online-only Table 1).

#### MySQL database

**Fig. 4:**
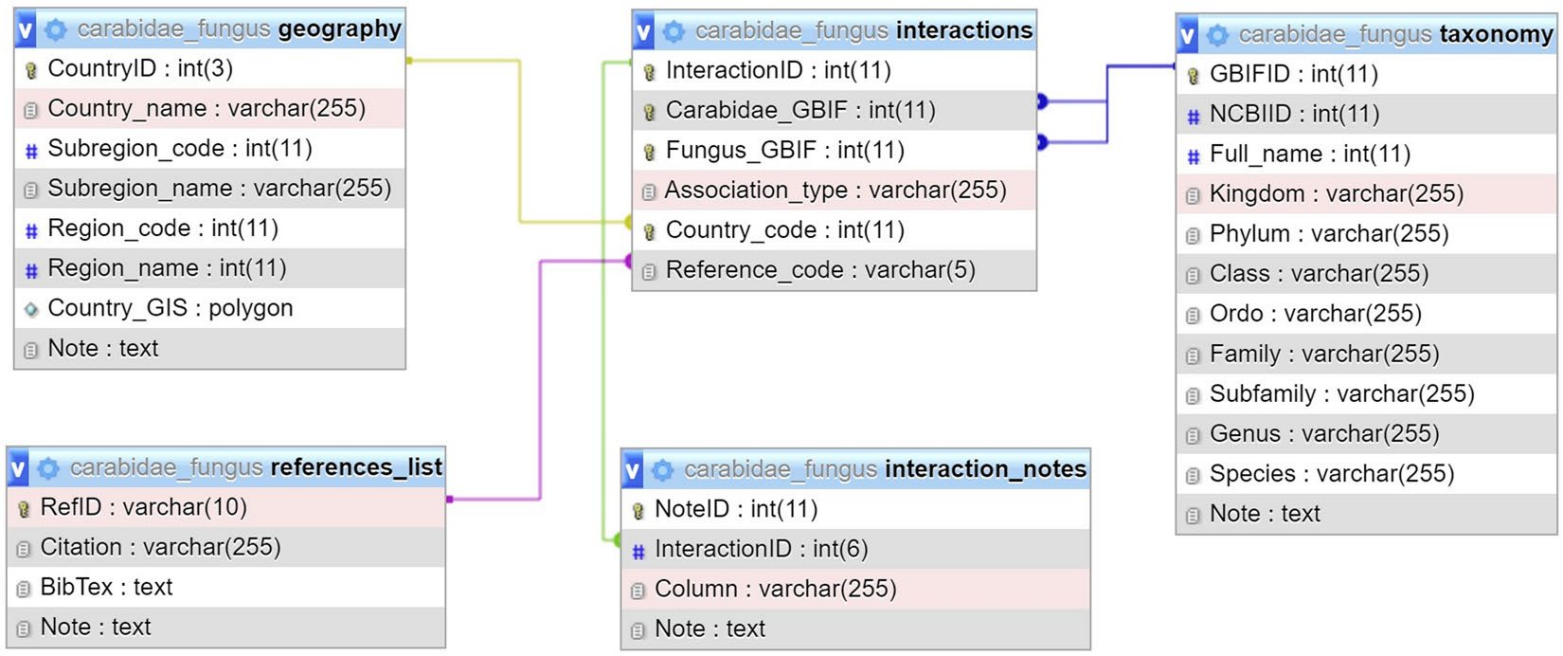
Structure of the MySQL database. Tables with data recording fields are shown and relationships between tables and linking data fields are marked with coloured lines.

A single.sql file has been provided for importing the entire database with a line of command, or with a few clicks, into an empty MySQL database. After importing, the aforementioned data tables and table links will be made visible (Fig. 4), and the database can be queried directly (e.g. through R).

#### Interactive web-interface

An interactive Shiny^31^ app has been provided on the http://54.252.94.194:3838/Carafun/ website to facilitate general data digestion, advanced filtering, and data visualisation.

## Technical Validation

The list of species was both programmatically cross-checked against taxonomic databases, and screened by experts in carabid and fungal taxonomy. Data with uncertain taxonomies (i.e. when resolving a reported taxon to a valid GBIF ID has failed) were excluded from the primary database but stored in a separate dataset. These uncertainties affected < 10% of the collected species names, and < 5% of the data. The authors validated association types, and questionable cases were determined by multi-expert consensuses. When country categorizations were unclear, either due to historical changes in boundaries or because of data recording incongruities, the original text was re-checked and, if provided, the country was re-confirmed based on the exact sampling location. Data with missing country records were still included in the main database and the most precise available location was given in a note. However, if an association with no geographic information was a duplicate of a more precise one, only the latter was retained and the other one deleted.

Each data point was also subjected to manual checks and all the edits in the database were recorded and stored as metadata. As a result of the structured relational database design, our database is easy to update, query and is convertible to other structures. Moreover, since the taxonomy in our database was carefully checked and unique identifiers from other major databases are assigned to each taxonomical entry, it can easily be linked to other international databases. Besides the data table format, the bibliographical information is available both in a human-readable citation format (APA style), and as a BibTex entry that facilitates several ways of usage. The database will also be maintained in the future, and will be made available for registered users to upload new data through a web-based graphical interface. New data will be curated, which will allow the database to be regularly expanded. Moreover, biotic interactions not found on GloBi’s server will be uploaded to that database as well.

## Data Availability

Computer codes used to generate and clean the Carabidae – Fungi database, as well as those to produce plots in the article and the Shiny app are freely available at https://github.com/pozsgaig/CaraFun.

## Online-only Table

**Online-only Table 1:** Data descriptors and the way how they are organized into tables for the three different data formats provided.

Column name	Column description	Single large data file	Associations data table	Taxonomy data table	References data table	Geography data table	Notes table
Carabidae_GBIFID	Unique identifier of Carabidae species and genera, the same as the identifier of taxa in the GBIF database. It is used to link Associations and Taxonomy data tables.	Y	Y	Y			
Carabidae_full_name	Human readable name of Carabidae species/genus. Author and year of description are also included.	Y		Y			
Fungus_GBIFID	Unique identifier of Fungi species and genera, the same as the identifier of taxa in the GBIF database. It is used to link Associations and Taxonomy data tables.	Y	Y	Y			
Fungus_full_name	Human readable name of Fungus species/genus. Author and year of description are also included.	Y		Y			
Inter_type	The type of interaction between a ground beetle and fungus species/genus	Y	Y				
Country_code/ CountryID	Unique identifier of a country, with some exceptions they are the same as the identifier of countries in the UN Geoscheme. It is used to link Associations and Geography data tables.	Y	Y			Y	
Country_name	Human readable country name.	Y				Y	
Country_abbreviation	ISO standard abbreviation of country names.	Y				Y	
RegionID/ Region_code	Unique identifier of a geographic region, with some exceptions they are the same as the identifier of geographic regions in the UN Geoscheme.	Y				Y	
Region_name	Human readable geographic region name.	Y				Y	
SubregionID/ Subregion_code	Unique identifier of a geographic subregion, with some exceptions they are the same as the identifier of subgeographic region in the UN Geoscheme.	Y				Y	
Subregion_name	Human readable geographic subregion name.	Y				Y	
ReferenceID	Unique identifier of a reference. It is used to link Associations and References data tables.	Y	Y		Y		
Short_citation	Human readable reference.	Y			Y		
Full_citation	Human readable HTML reference.	Y			Y		
BibTex	Full citation in a computer readable BibTex format	Y			Y		
Carabidae_NCBIID	Optional identifier of the Carabidae species/genus to the NCBI database	Y		Y			
Carabidae_rank	The lowest taxonomical rank a Carabidae taxon was resolved to	Y		Y			
Carabidae_kingdom	Carabidae taxonomy	Y		Y			
Carabidae_phylum		Y		Y			
Carabidae_class		Y		Y			
Carabidae_order		Y		Y			
Carabidae_family		Y		Y			
Carabidae_subfamily		Y		Y			
Carabidae_genus		Y		Y			
Carabidae_species		Y		Y			
Fungus_NCBIID	Optional identifier of the Carabidae species/genus to the NCBI database	Y		Y			
Fungus_rank	The lowest taxonomical rank a fungus taxon was resolved to	Y		Y			
Fungus_kingdom	Fungal taxonomy	Y		Y			
Fungus_phylum		Y		Y			
Fungus_class		Y		Y			
Fungus_order		Y		Y			
Fungus_family		Y		Y			
Fungus_subfamily		Y		Y			
Fungus_genus		Y		Y			
Fungus_species		Y		Y			
Carabidae_note	Additional notes related to Carabidae taxonomy.	Y					Y
Fungus_note	Additional notes related to Fungus taxonomy.	Y					Y
Association_note	Additional notes related to the interaction.	Y					Y
Country_note	Additional notes related to recorded location.	Y					Y
Ref_note	Additional notes related to the reference.	Y					Y
