# Author Correction: Associations between carabid beetles and fungi in the light of 200 years of published literature

**DOI:** 10.1038/s41597-022-01404-4

**Published:** 2022-05-30

**Authors:** Gábor Pozsgai, Ibtissem Ben Fekih, Markus V. Kohnen, Said Amrani, Sándor Bérces, Dávid Fülöp, Mohammed Y. M. Jaber, Nicolai Vitt Meyling, Malgorzata Ruszkiewicz-Michalska, Walter P. Pfliegler, Francisco Javier Sánchez-García, Jie Zhang, Christopher Rensing, Gábor L. Lövei, Minsheng You

**Affiliations:** 1 State Key Laboratory of Ecological Pest Control for Fujian and Taiwan Crops, Institute of Applied Ecology, Fujian Agriculture and Forestry University, Fuzhou, 350002 China; 2 Joint international Research Laboratory of Ecological Pest Control, Ministry of Education, Fuzhou, 350002 China; 3 CE3C – Centre for Ecology, Evolution and Environmental Changes, Azorean Biodiversity Group and Universidade dos Açores, Angra do Heroísmo, 9700-042 Azores, Portugal; 4 Institute of Environmental Microbiology, College of Resources and Environment, Fujian Agriculture and Forestry University, Fuzhou, 350002 China; 5 Basic Forestry and Proteomics Research Center, College of Life Science, Fujian Provincial Key Laboratory of Haixia Applied Plant Systems Biology, Fujian Agriculture and Forestry University, Fuzhou, 350002 China; 6 Laboratoire de Biologie et de Physiologie des Organismes, Faculté des Sciences Biologiques, Université des Sciences et de la Technologie Houari Boumediène, BP 32 El Alia, Alger, 16111 Algeria; 7 Duna-Ipoly National Park Directorate, Költő u. 21, H-1121 Budapest, Hungary; 8 Juhász-Nagy Pál Doctoral School, University of Debrecen, Egyetem tér 1, H-4032 Debrecen, Hungary; 9 Department of Zoology, Plant Protection Institute, Centre for Agricultural Research, Nagykovácsi út 26-30, H-1029 Budapest, Hungary; 10 Fujian University Key Laboratory for Plant-Microbe Interaction, College of Plant Protection, Fujian Agriculture and Forestry University, Fuzhou, 350002 China; 11 Department of Plant and Environmental Sciences, University of Copenhagen, Thorvaldsensvej 40, 1871 Frederiksberg C, Denmark; 12 Department of Algology and Mycology Faculty of Biology and Environmental Protection, University of Łódź, Banacha 12/16, PL-90-237 Łódź, Poland; 13 Department of Molecular Biotechnology and Microbiology, University of Debrecen, Egyetem tér 1, Debrecen, H-4032 Hungary; 14 Fujian Provincial Key Laboratory of Insect Ecology, College of Plant Protection, Fujian Agriculture and Forestry University, Fuzhou, 350002 China; 15 Área de Biología Animal, Departamento de Zoología y Antropología Física, Facultad de Veterinaria, Universidad de Murcia, Murcia, 30100 Spain; 16 Department of Agroecology, Aarhus University, Flakkebjerg Research Centre, Forsøgsvej 1, DK-4200 Slagelse, Denmark

**Keywords:** Agroecology, Macroecology, Ecological networks

Correction to: *Scientific Data* 10.1038/s41597-021-01072-w, published online 04 November 2021

The original version of this Data Descriptor implied that the Global Biotic Interactions database (GloBi) was a sub-site of the Global Biodiversity Information Facility (GBIF) which is incorrect. It also implied that data sources relating to literature references and location information were unavailable in GloBi, whereas these can retrieved by changing the parameters in the get_interactions() function of the rglobi R package. The text in the Methods section has been amended to reflect this.

